# Acquired Thrombotic Thrombocytopenic Purpura in a Newly Diagnosed HIV Patient: A Case Report and Literature Review

**DOI:** 10.7759/cureus.15967

**Published:** 2021-06-27

**Authors:** Mohsen S Alshamam, Vikram Sumbly, Saifullah Khan, Nso Nso, Vincent Rizzo

**Affiliations:** 1 Internal Medicine, Icahn School of Medicine at Mount Sinai, New York City Health and Hospitals/Queens, NYC, USA; 2 General Medicine, Saint James School of Medicine, St. Vincent, VCT

**Keywords:** thrombotic thrombocytopenic thrombocytopenia, hiv aids, acquired ttp, anemia, schistocytes

## Abstract

Thrombotic thrombocytopenic purpura (TTP) is a rare but a potentially fatal condition. Although the majority of TTP cases are of unknown etiology, certain viral infections, malignancies, and medications have been linked to the acquired form of the illness. Regardless of the underlying etiology, TTP remains a great challenge diagnostically and therapeutically. TTP remains a very uncommon complication of HIV. We reviewed the current literature to better understand the relationship between HIV and TTP and address some of the major obstacles that may impede or delay the correct diagnosis. Here, we present a case of a 28-year-old male with complaints of light-headedness, fatigue, and gingival bleeding. He was found to have severe anemia and thrombocytopenia. He tested positive for the HIV and was then diagnosed with TTP. Despite needing endotracheal intubation for airway protection, he clinically improved with packed red blood cells, plasmapheresis, and highly active antiretroviral therapy.

## Introduction

With an approximate annual incidence of one per 1,000,000 of the population, thrombotic thrombocytopenic purpura (TTP) is a rare and a serious subtype of thrombotic microangiopathy [1]. This hematological syndrome is characterized by the formation of systemic thrombosis due to the premature activation of the coagulation cascade and the spontaneous aggregation of platelets. This process is initiated by the abnormal accumulation of the von Willebrand Factor (VWF) within the circulatory system due to the deficiency of the ADAMTS13 metalloprotease [2]. TTP has been linked to rheumatological conditions, malignancies, infections, and medications [3]. Although the pathogenesis of HIV-induced TTP remains poorly understood, this virus has been shown to increase the risk of acquiring TTP by 15-40 times [4]. An HIV patient’s comorbidities, CD4 count, and response to treatment are all factors that affect the overall prognosis. Here, we present the case of a 28-year-old male who developed TTP after being diagnosed with HIV.

## Case presentation

A 28-year-old male with no significant past medical history presented to the emergency department (ED) complaining of exertional light-headedness, fatigue, and intermittent gingival bleeding for the last two weeks. He also experienced an unintentional 20 lbs weight loss over the last two months and exertional chest discomfort. Otherwise, he denied any subjective fever, chills, headaches, palpitations, cough, abdominal pain, nausea, vomiting, diarrhea, bright red blood per rectum, melena, dysuria, hematuria, arthralgia, skin changes, or rashes. He drinks occasionally but denies any tobacco or illicit drug use. He is sexually active with males.

The initial workup in the ED revealed severe normocytic anemia, severe thrombocytopenia, and an elevated reticulocyte count (Table 1). A sexually transmitted disease screening test was positive for HIV, which was then confirmed with a repeat test. The patient’s viral load was elevated and his cluster of differentiation-4+ (CD-4+) T-cells were low (Table 2). His electrocardiogram (ECG) was normal sinus rhythm and his chest x-ray returned negative for any acute pathological process. Further workup was significant for elevated lactate dehydrogenase, creatine kinase, d-dimer, and decreased haptoglobin (Tables 1 and 2). The blood smear was significant for 7-12 schistocytes per multiple high-power fields, polychromasia, nucleated red blood cells (RBCs), paucity of platelets, dysplastic neutrophils, and normal/atypical lymphocytes.

**Table 1:** Labs that were monitored frequently include initial and final (at discharge) *Approximately one month after discharge.

Lab Name	Initial Value	Final Value	Reference Range
Hemoglobin	6.1	11	13.5-17.5 g/dL
Hematocrit	19	33.2	41-53%
Mean corpuscular volume	96	93	83-98 fL
White blood cells	10.33	8.14	3.9-10.6/nL
Platelets	12	388	150-440/nL
Reticulocyte count	0.2540	0.0923	0.0500-0.1000 cells/pL
Blood urea nitrogen	19	21	5-26 mg/dL
Creatinine	1.04	0.98	0.5-1.5 mg/dL
Haptoglobin	<20	261	34-200 mg/dL
Lactate dehydrogenase	1219	260	100-210 U/L
ADAMTS-13 activity	<2.0	12.8 and 69.4*	>66.8 %

**Table 2:** Other labs

Other Labs	Value	Reference Range
HIV-1 viral load	268622	Not detected
Absolute cluster of differentiation-4 count	333	489-1457/uL
Creatine phosphokinase	1232	20-190 U/L
D-dimer	1269	0-230 ng/mL
Ferritin	>2000	30-400 ng/mL
Vitamin B12	323	232-1245 pg/mL
Direct antiglobulin test	Negative	Negative
Fibrinogen	215	200-393 mg/dL
International normalized ratio/prothrombin time	12.2/1.1	10-13 seconds
Activated partial thromboplastin time	29.2	25.1-36.5 seconds

Given his anemia and thrombocytopenia with schistocytes concerning for hemolytic anemia, there was a growing concern for TTP despite the lack of fever, kidney dysfunction, and neurologic involvement. The following tests were obtained per hematology recommendations: a disintegrin and metalloproteinase with a thrombospondin type 1 motif member 13 (ADAMTS-13) activity, complete anemia workup, fibrinogen level, hemoglobin electrophoresis, and a direct antiglobulin test (DAT). Infectious disease consultation recommended a more thorough infectious workup (reported below) to determine the need for prophylaxis and starting the highly active antiretroviral therapy (HAART). Diagnostic tests of chlamydia, gonorrhea, syphilis, parvovirus B19, and cytomegalovirus were negative. The urine, stool, and blood culture did not grow any pathogens.

The patient was then transferred to the step-down unit as there was a growing concern for TTP. After a central line placement for imminent plasmapheresis, the patient exhibited signs of impaired level of consciousness and had to be intubated in the medical intensive care unit for airway protection. A basic metabolic profile was significant for acute renal failure. Fibrinogen, DAT, hemoglobin electrophoresis, urine toxicology, and prothrombin time (PT)-international normalized ratio (INR)/partial thromboplastin time (PTT) were normal. A low ADAMTS-13 activity confirmed a diagnosis of TTP. The patient was managed with plasmapheresis, methylprednisolone, blood product transfusions (packed red blood cells), B9/B12, HAART, ciprofloxacin, and metronidazole (given reported diarrhea). The patient was extubated once his clinical condition improved and was then sent back to the medical floor for further management. He was discharged shortly afterward with plans of following up at his outpatient clinic.

## Discussion

First described in 1987 by Jokela et al., HIV-induced TTP is an unusual syndrome that is characterized by RBC shearing and ischemic organ dysfunction due to the uncontrolled microvascular clumping of platelets [5]. While TTP is typically defined by the classic pentad of fever, microangiopathic hemolytic anemia, thrombocytopenia, renal failure, and transient neurological symptoms, most patients lack one or more of these clinical findings [6]. It is believed that a minority of TTP cases are caused by a known agent, whereas the remaining cases are idiopathic in nature [7]. The exact pathogenic mechanism of HIV-induced TTP has yet to be fully elucidated [7].

Upon our patient’s admission, the differential diagnoses included hemolytic uremic syndrome (HUS), Evans syndrome, disseminated intravascular coagulation (DIC), and heparin-induced thrombocytopenia (HIT). A diagnosis of HIT and Evans syndrome was unlikely because the patient never received any heparin, and his DAT was negative. DIC was also ruled out as his fibrinogen level, PTT, and PT-INR were within normal limits. HUS was a strong consideration, but the patient did not experience any episodes of bloody/non-bloody diarrhea before hospitalization and his stool culture was negative for *Escherichia coli* O157:H7. It was the abnormally low ADAMTS-13 activity assay that favored a diagnosis of TTP.

It is hypothesized that it is the combination of endothelial damage, infectious stress, and decreased ADAMTS-13 activity secondary to the abnormal production of anti-ADAMTS-13 antibodies that causes TTP in HIV patients [8,9]. These abnormalities facilitate the formation of ultra-large vWF multimers that bind to damaged endothelial cells and platelets [9]. These newly formed vWF-platelet aggregates will then embolize, shear RBCs, and occlude smaller vessels [9]. Our patient’s thrombocytopenia was caused by his platelets being “consumed” by large vWF multimers, while his microcytic anemia was a result of direct RBC hemolysis. His renal injury and neurological symptoms were likely ischemic in nature secondary to systemic microvascular thromboembolic events.

Acute episodes of TTP are often well controlled with plasmapheresis and glucocorticoid therapy [9]. Unfortunately, relapse is a common occurrence as these interventions do not eliminate the source of the disease - the anti-ADAMTS-13 antibody-producing B cells [9]. Studies have shown that patients started on rituximab have a reduced risk of disease recurrence because this medication has been shown to suppress autoantibody production [10,11]. For patients with rituximab-refractory TTP, bortezomib has shown promise as salvage therapy [12].

There were several reasons as to why our patient’s TTP diagnosis was delayed, and which put him at risk for further hematological complications. TTP was initially not considered as a top differential diagnosis for several reasons: (1) the patient’s anemia was believed to be a separate entity, not related to an ongoing hemolytic process; (2) the lack of fever, renal injury, or neurological symptoms on admission; (3) the lack of many schistocytes on the first manual differential. It is the combination of these events that prevented an early diagnosis of TTP and rapid initiation of plasmapheresis/glucocorticoid therapy. Therefore, TTP should always be kept in mind in HIV patients unexplainably developing severe anemia and thrombocytopenia. This can drastically decrease morbidity and mortality.

## Conclusions

HIV-related TTP is a rare disorder that may be fatal if not properly diagnosed. Even with the absence of schistocytes on a peripheral smear and the classic pentad of symptoms, a high suspicion for TTP should be maintained for anyone with unexplained severe anemia and thrombocytopenia. This will protect patients from further TTP complications as the management of this disease is time- and effort dependent. 
